# Dosimetric impact of intrafraction motion on boosts on intraprostatic lesions: a simulation based on actual motion data from real time ultrasound tracking

**DOI:** 10.1186/s13014-019-1285-1

**Published:** 2019-05-16

**Authors:** Hendrik Ballhausen, Minglun Li, Michael Reiner, Claus Belka

**Affiliations:** Department of Radiation Oncology, University Hospital, LMU Munich, Marchioninistrasse 15, 81377 Munich, Germany

**Keywords:** Prostate cancer, Intrafraction motion, Intraprostatic lesion, Simultaneous integrated boost, Dosimetry, Ultrasound

## Abstract

**Background:**

Intrafraction motion is particularly problematic in case of small target volumes and narrow margins. Here we simulate the dose coverage of intraprostatic lesions (IPL) by simultaneous integrated boosts (SIB). For this purpose, we use a large sample of actual intrafraction motion data.

**Methods:**

Fifty-three h of intra-fraction motion of the prostate were recorded in real-time by 4D ultrasound (4DUS) during 720 fractions in 28 patients. We simulate spherical IPLs with 3, 5, and 7 mm radius and matching spherical SIBs with 0, 2, and 5 mm safety margins. The volumetric overlap between IPLs and SIBs is calculated. Dose volume histograms (DVH) are estimated by Monte Carlo simulation.

**Results:**

On average, the distance of the prostate was 1.3 mm from its initial position over all fractions and patients. Average volumetric overlap was 73, 82, and 87% of IPL volume in case of 3, 5, and 7 mm IPLs and SIBs without safety margins. These improved to 95% or more in case of 2 mm safety margins and 98% or more in case of 5 mm safety margins. DVHs showed that 80% of the IPL volume received 60, 72, and 79% of maximum dose in case of 3, 5, and 7 mm IPLs and SIBs without safety margins. These improved to 94% or more given moderately sized safety margins of 2 mm.

**Conclusions:**

On average over all fractions and patients, the dose coverage would have been acceptable even for small target volumes such as IPLs of radius 3 to 7 mm and narrow fields. Moderate safety margins of 2 mm could have ensured a delivery of 90% or more of the SIB dose to the IPL. In this case, SIB volume would have been considerably larger than IPL volume, but still considerably smaller than the overall PTV of the prostate.

**Electronic supplementary material:**

The online version of this article (10.1186/s13014-019-1285-1) contains supplementary material, which is available to authorized users.

## Background

Recurrence after radiotherapy of prostate carcinoma often originates from a macroscopic intraprostatic lesion (IPL). A simultaneous integrated boost (SIB) to the dominant IPL is a strategy to fight such recurrences [1–4]. Toxicity seems to be less of a problem [5–8], however it has been suggested that intrafraction motion requires either intrafraction motion tracking in case of small margins [9] or substantial margins [10].

Generally, the recording of the prostate position by 4D ultrasound [11] or kV-imaging [12, 13] during a fraction has received some interest in recent years and is a natural extension of these techniques from inter-fraction position verification and correction to online intra-fraction monitoring and adaption. The hope is, e.g. to account for prostate intra-fraction motion by gating the beam or tracking the prostate by continually adjusting the multi-leaf collimator [ 14–17].

In this simulation study we calculate the impact of intrafraction motion on the volumetric coverage between IPL and SIB and on dose volume histograms (DVH). We use motion data from real time ultrasound tracking during actual fractions and apply this data to idealized spherical IPL volumes of 3, 5, and 7 mm radius and to idealized spherical SIB volumes without and with safety margins of 2 mm resp. 5 mm.

Our main question is, if and what size of margins is required to counterbalance intrafraction motion and ensure an acceptable dose coverage of the IPL.

## Methods

### Intrafraction motion data

The analysis is based on the same set of patients and raw data as reported in [18]. The study is based on 28 patients with adenocarcinoma of the prostate who received a definitive external beam radiotherapy between June 2014 and March 2017 at our department. The 28 patients were of a typical demographics for prostate cancer, as were the treatment regimes, see Table 1.

**Table 1:** Patient demographics and treatment regimes

Number of patients	28
Fractions recorded	720
Duration recorded	53 h 32 m 59 s
Age (years)	median: 72.4 range: 53.2 to 85.9
Body size (cm)	median: 178 range: 163 to 195
Body weight (kg)	median: 82 range: 63 to 106
Prostate volume (ml)	median: 45 range: 15 to 90
T (%)	T1–13 (46%) T2–10 (36%) T3–5 (18%)
Number of fractions	35–1 (4%) 36–5 (18%) 37–10 (36%) 38–12 (43%)
Total dose (Gy)	median: 74.0 avg. ± std.: 74.4 ± 1.7 range: 70.0 to 76.0

Up to the calculation of prostate trajectories, the raw data and methods were identical to those detailed in [18].

Vertical (anterior-posterior), longitudinal (cranial-caudal), lateral (left-right), and radial (‘vector length’ or Euclidean ‘3D distance’) prostate displacements were calculated relative to the starting position of the prostate (as recorded by 4DUS) after image guide repositioning (by kV-CBCT) at the beginning of the respective fraction.

Averages, standard deviations, quantiles (5, 25%, median, 75, and 95%) and root mean squares (r.m.s.) of vertical, longitudinal, lateral, and radial displacements were calculated.

### Volumetric coverage

The IPL was idealized as a sphere of radius r. The dose distribution was idealized as constant within a spherical region of radius r + m and zero outside, where m denotes the safety margin. These idealizations were assumed for simplicity, and because they are not uncommon, e.g. in optimal margin recipes.

The 3D-distance or Euclidean distance d of the prostate from the origin determined the distance of the centers of these two spheres. The volume of the overlap of the two spheres in case m < d < r + m was.

$$ {\mathrm{V}}_{\mathrm{overlap}}=\uppi /\left(12\mathrm{d}\right)\ast {\left(2\mathrm{r}+\mathrm{m}-\mathrm{d}\right)}^2\ast \left({\mathrm{d}}^2+2\mathrm{d}\left(2\mathrm{r}+\mathrm{m}\right)-3\;{\mathrm{m}}^2\right) $$

See Fig. 1.

**Fig. 1:**
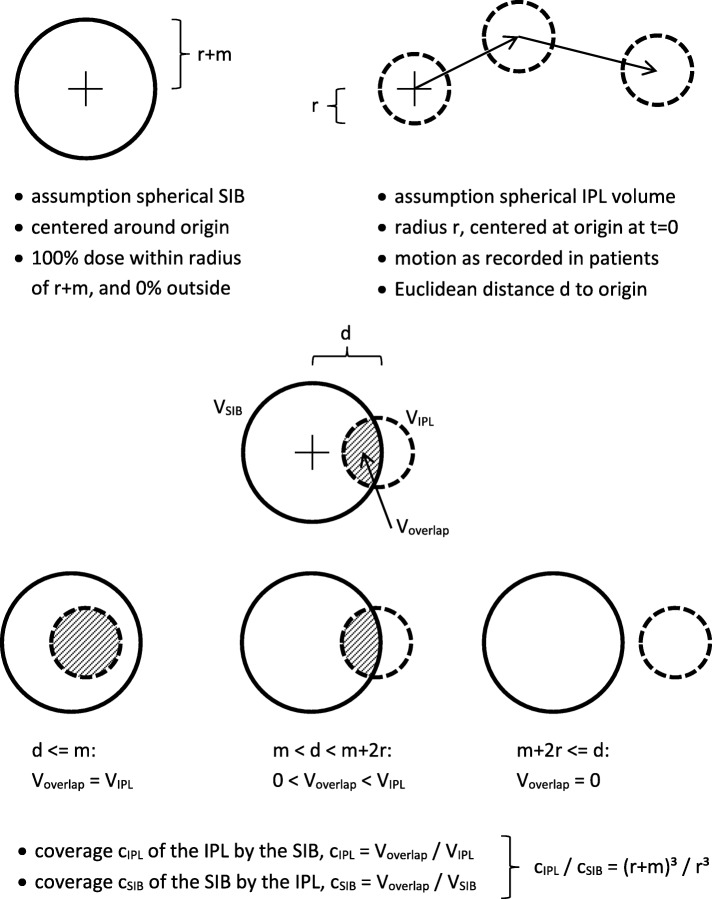
Calculation of the overlap between SIB dose and IPL volume

This volume was divided by the volume of the IPL, V_IPL_ = 4π/3 * r^2^ to arrive at the coverage.

$$ {\displaystyle \begin{array}{cc}{\mathrm{c}}_{\mathrm{IPL}}=100\%& \mathrm{in}\ \mathrm{case}\ \mathrm{d}<=\mathrm{m}\\ {}{\mathrm{c}}_{\mathrm{IPL}}=0\%& \mathrm{in}\ \mathrm{case}\ \mathrm{d}>=2\mathrm{r}+\mathrm{m}\\ {}{\mathrm{c}}_{\mathrm{IPL}}={\mathrm{V}}_{\mathrm{overlap}}/{\mathrm{V}}_{\mathrm{IPL}}& \mathrm{in}\ \mathrm{case}\ \mathrm{m}<\mathrm{d}<2\mathrm{r}+\mathrm{m}\end{array}} $$

This coverage c_IPL,i_ was calculated for every single one of the 39,295 measured 3D-distances d_i_ and for every combination of IPL radius r (3 mm, 5 mm, 7 mm) and safety margin m (0 mm, 2 mm, 5 mm). Finally, the respective averages, standard deviations, quantiles (5, 25%, median, 75, and 95%) over all measurements were calculated.

The aforementioned coverage of the IPL volume by the SIB volume, in % of IPL volume, concerns the question how much dose was received by the IPL. The other interesting question is how much of the overall SIB dose was received by the IPL. To this end, the coverage c_SIB_ of the larger SIB volume by the smaller IPL volume, in % of SIB volume, was also calculated. This ‘reverse’ calculation yields:

$$ {\displaystyle \begin{array}{cc}{\mathrm{c}}_{\mathrm{SIB}}={\mathrm{r}}^3/{\left(\mathrm{r}+\mathrm{m}\right)}^3& \mathrm{in}\ \mathrm{case}\ \mathrm{d}<=\mathrm{m}\\ {}{\mathrm{c}}_{\mathrm{SIB}}=0\%& \mathrm{in}\ \mathrm{case}\ \mathrm{d}>=2\mathrm{r}+\mathrm{m}\\ {}{\mathrm{c}}_{\mathrm{SIB}}={\mathrm{V}}_{\mathrm{overlap}}/{\mathrm{V}}_{\mathrm{SIB}}& \mathrm{in}\ \mathrm{case}\ \mathrm{m}<\mathrm{d}<2\mathrm{r}+\mathrm{m}\\ {}\mathrm{Generally},{\mathrm{c}}_{\mathrm{SIB}}={\mathrm{r}}^3/{\left(\mathrm{r}+\mathrm{m}\right)}^3{\mathrm{c}}_{\mathrm{IPL}.}& \end{array}} $$

### Dose volume histograms

For the calculation of DVHs, 5000 points within the IPL sphere of radius r were randomly and uniformely sampled (Monte Carlo). The points were parametrized as a triple (a, b, c). For each measured position (x_i_,y_i_,z_i_) of the prostate, it was checked if (a + x_i_)^2^ + (b + y_i_)^2^ + (c + z_i_)^2^ < = (r + m)^2 lay within the sphere covered by the SIB. If so, a unit of dose was added, and the received units of dose were divided by the number of samples, 39,925. Each of these dose percentages was then added to the histogram of 5000 such dose percentages which was plotted as the DVH. Again, this procedure was done for every combination of IPL radius r (3 mm, 5 mm, 7 mm) and safety margin m (0 mm, 2 mm, 5 mm).

Data handling, statistical analysis and plots were performed with Microsoft Excel and VBA.

## Results

### Intrafraction motion data

On average over all patients and fractions, the prostate was displaced by about 1.3 mm radially from its initial position. In some cases, the prostate drifted off by 1 cm or more, but during 95% of the time, it remained within 4.3 mm of its initial position, see Table 2.

**Table 2:** Recorded displacements of the prostate from its initial position

	Longitudinal displacement	Lateral displacement	Vertical displacement	Euclidean 3D distance
Average	−0.13	0.00	0.11	1.28
Std.dev.	1.15	0.76	1.64	1.72
Minimum	−13.11	−11.43	−7.40	0.00
5% quantile	−1.87	−0.94	−1.78	0.12
25% quantile	−0.27	−0.19	− 0.51	0.37
Median	0.02	0.00	−0.10	0.73
75% quantile	0.30	0.22	0.34	1.49
95% quantile	1.15	0.90	2.88	4.26
Maximum	6.03	8.87	17.52	21.20

Displacements of the prostate from its initial position along different axes. All units are mm

### Volumetric coverage

Even without safety margins, a same-sized SIB would have covered 73, 82% resp. 87% of the volume of an IPL of radius 3 mm, 5 mm resp. 7 mm, on average. 95% of the time, it would have covered at least 11, 40% resp. 56% of the IPL volume. These numbers improve substantially for a moderately sized safety margin of 2 mm. In this case, average coverage would have been at least 95, and 95% of the time (and hence, dose) would have been applied to a majority of the IPL volume. Furthermore, with 5 mm safety margin, average coverage would be around 99, and 95% of the time the SIB would have completely covered the IPL volume. See Table 3.

**Table 3:** Volumetric coverage c_IPL_ of the IPL by the SIB, in % of IPL volume

Safety margin	none			2 mm			5 mm		
IPL radius	3 mm	5 mm	7 mm	3 mm	5 mm	7 mm	3 mm	5 mm	7 mm
Average	73%	82%	87%	95%	96%	97%	98%	99%	99%
Std.dev.	25%	19%	15%	18%	14%	11%	11%	8%	6%
Minimum	0%	0%	0%	0%	0%	0%	0%	0%	0%
5% quantile	11%	40%	56%	57%	76%	84%	100%	100%	100%
25% quantile	63%	78%	84%	100%	100%	100%	100%	100%	100%
Median	82%	89%	92%	100%	100%	100%	100%	100%	100%
75% quantile	91%	95%	96%	100%	100%	100%	100%	100%	100%
95% quantile	97%	98%	99%	100%	100%	100%	100%	100%	100%
Maximum	100%	100%	100%	100%	100%	100%	100%	100%	100%

Percentage c_IPL_ of the IPL volume that is covered by the SIB volume

Safety margins, however, would also have had a downside in terms of dose efficiency resp. potentially toxicity. Due to the cubic power in the ratio between IPL volume (proportional to r^3) and SIB volume (proportional to (r + m)^3) the ratio would have fallen off quickly even in the best case of completely overlapping spheres. For example, in case of a 2 mm margin for a 5 mm radius lesion, the volume of the IPL would have been only 36% of the SIB, with two thirds of the boost dose going to surrounding non-lesion prostate tissue or even non-prostate tissue. The percentage of SIB dose that would have gone to the IPL is shown in Table 4 for the different situations.

**Table 4:** Volumetric coverage c_SIB_ of the SIB by the IPL, in % of SIB volume

Safety margin	none			2 mm			5 mm		
IPL radius	3 mm	5 mm	7 mm	3 mm	5 mm	7 mm	3 mm	5 mm	7 mm
Average	73%	82%	87%	20%	35%	46%	5%	12%	20%
Std.dev.	25%	19%	15%	4%	5%	5%	1%	1%	1%
Minimum	0%	0%	0%	0%	0%	0%	0%	0%	0%
5% quantile	11%	40%	56%	12%	28%	40%	5%	13%	20%
25% quantile	63%	78%	84%	22%	36%	47%	5%	13%	20%
Median	82%	89%	92%	22%	36%	47%	5%	13%	20%
75% quantile	91%	95%	96%	22%	36%	47%	5%	13%	20%
95% quantile	97%	98%	99%	22%	36%	47%	5%	13%	20%
Maximum	100%	100%	100%	22%	36%	47%	5%	13%	20%

Percentage c_SIB_ of the SIB volume that is covered by the IPL volume

### Dose volume histograms

**Fig. 2:**
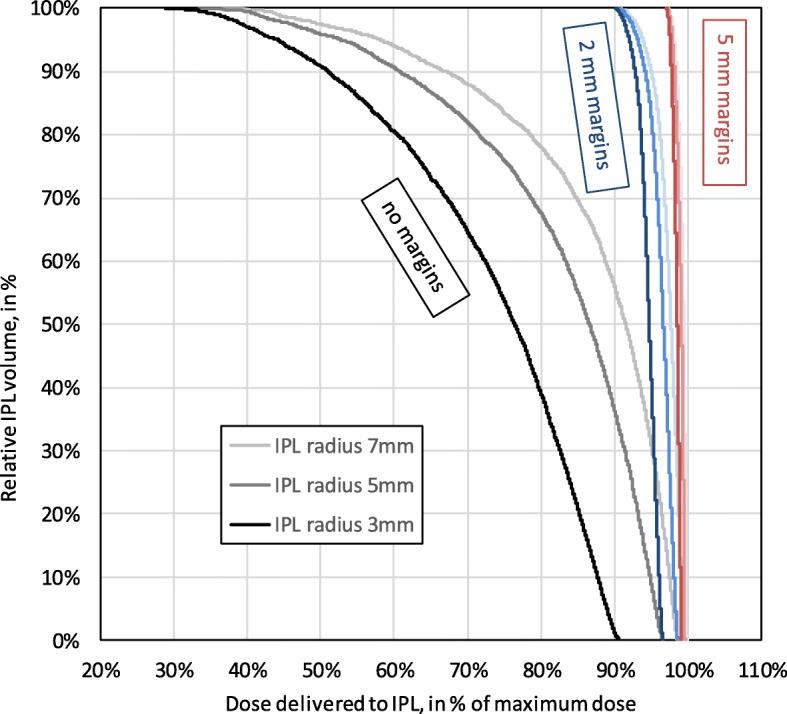
Dose volume histograms. Cumulative histogram of the dose volume relationship with dose on the horizontal axis (in percentage of maximum dose) and volume on the vertical axis (in percentage of IPL volume). Result of Monte Carlo simulations for different combinations of IPL radius (3 5 and 7 mm) and SIB safety margin (0 2 and 5 mm)

The dose volume histograms draw a slightly more differentiated picture, see Fig. 2.

In all situations, 30% of the dose would have been received by 100% of the IPL volume, with our without safety margins.

Without safety margins, 50% of the dose would have been received by 91, 96% resp. 98% of the IPL volume of radius 3 mm, 5 mm resp. 7 mm. With safety margins of either 2 mm or 5 mm, that 50% dose would have been received by 100% of the IPL volume.

At 95% of dose, the size of the safety margin begins to matter. An IPL volume of 3 mm receives 0% (no safety margins), 42% (2 mm safety margin) resp. 100% (5 mm safety margin) of the dose. Similarly, the dose received by an IPL volume of 5 mm increases from 9% (no safety margin) to 83% (2 mm) to 100%. In case of an IPL volume of 7 mm, this receives already 31% of dose without any safety margins, and this value increases to 90% in case of 2 mm margins. See in the Additional file 1: Table S5.

Conversely, e.g. 95% of the IPL volume of radius 3 mm, 5 mm resp. 7 mm receive 44, 53% resp. 58% of the nominal SIB dose in the absence of safety margins. This percentage rises to over 90% in case of 2 mm margins and to 98% in case of 5 mm margins. These figures can be recovered by scaling the earlier results by (r + m)^3^/r^3^, as explained above.

### Patient variability

**Fig. 3:**
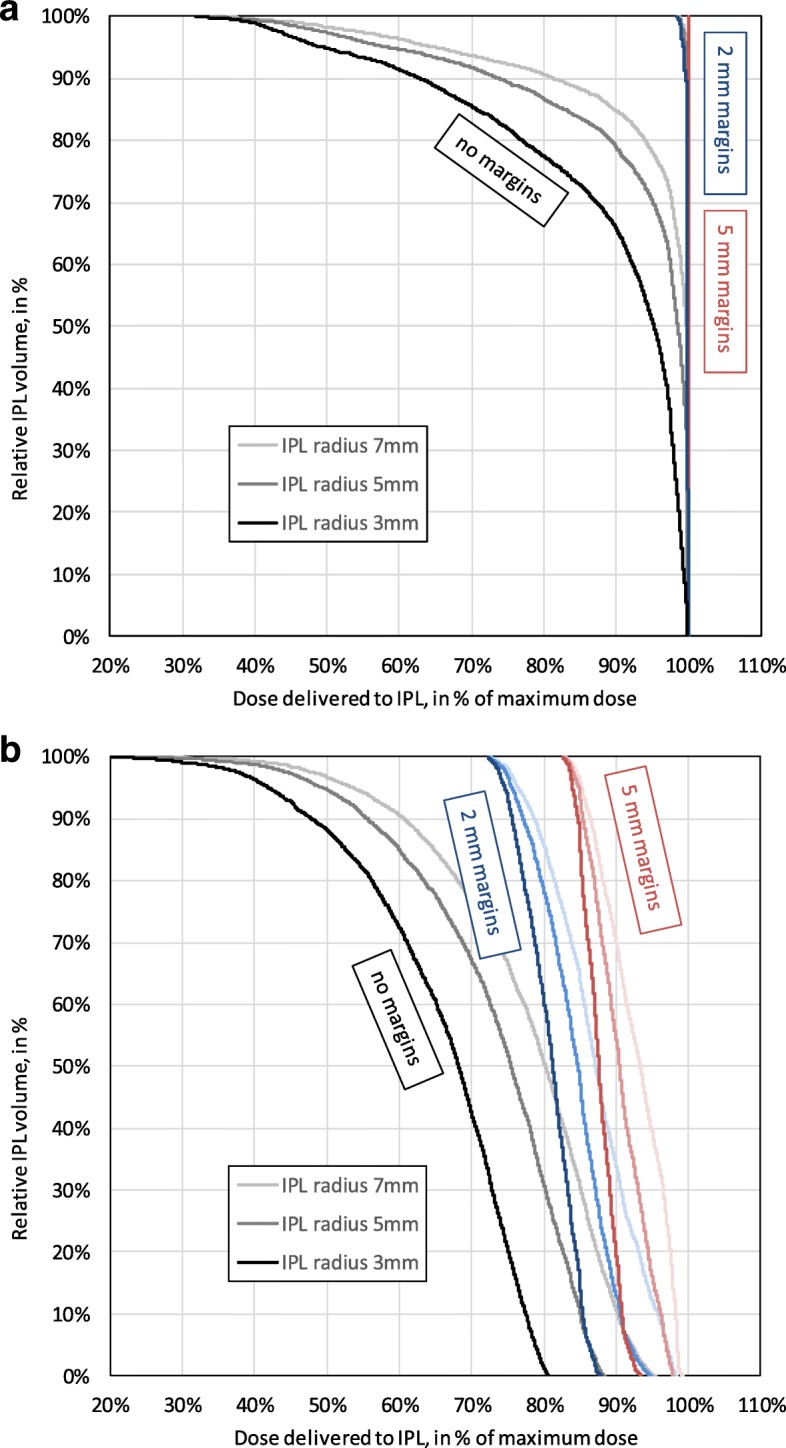
Dose volume histograms for individual patients (extreme cases of minimal and maximal motility). As Fig. 1 but for individual patients. **a** The top plot shows the DVHs for patient #13 who featured a particular small average radial displacement of the prostate of 0.50 mm. **b** By contrast the bottom plot shows the DVHs for patient #11 with a particularly large average radial displacement of the prostate of 2.53 mm

The motility of the prostate differs in patients. While the average radial displacement of the prostate was 1.28 mm across all patients and fractions, it was as small as 0.50 mm in one patient (patient #13) and as large as 2.53 mm in another (patient #11). The median was 1.06 mm with quartiles 0.75 mm and 1.39 mm. Similarly, the maximum radial displacement of the prostate was only 2.68 mm in one patient (patient #27) and as large as 21.20 mm in another (patient #10). The median was 7.82 mm with quartiles 4.96 mm and 10.07 mm. These differences translate into the DHVs. To illustrate the two extreme cases, Fig. 3 shows the DVHs for patients #13 and #11 side by side:

## Discussion

A recent review on IPL boosts [3] reports a mean of 2.4 cm^3^ for the mean/median IPL volumes reported by multiple studies. Due to the nonlinear relationship between radius and volume, this corresponds to a mean IPL radius probably well below 8 mm. In our simulation we use a cautious assumption of 3, 5, and 7 mm in order to highlight the impact of intrafraction motion on smaller targets.

Sing et al. [1] found that four IPLs in three patients were successfully targeted with a 3 mm margin. In our simulation, using a margin of 2 mm or more ensures at least 90% of prescribed dose to the entire IPL volume.

A large and recent study [7] reports that 284 patients with an additional SIB of 95 Gy to the IPL did not differ in toxicity from the base group of 287 patients receiving only 77 Gy to the entire prostate. Other studies show similar levels of tolerance [5, 6, 8]. Our data shows that the average displacement of the prostate is 1.3 mm. 95% of the time, the prostate remains within 4.3 mm of its initial position, notably without any mitigation (such as online correction of the table position or the multi leaf collimator). While this means that the SIB is not always centered on the IPL, the SIB will hit prostate tissue predominantly, depending on the location of the IPL within the prostate. Our simulation indicates that the IPL would receive the majority of the SIB in case of an IPL radius of at least 8 mm and a safety margin of at most 2 mm. Without safety margin (reducing the coverage of the IPL by the SIB), the coverage of the SIB by the IPL rises to 73% resp. 87% in case of an IPL radius of 3 mm resp. 7 mm.

In a planning study with and without simulated intrafraction motion [10] a safety margin of 7 mm was found to make both sequential or simultaneous boosts feasible. This is in agreement with our simulation showing that already a safety margin of 5 mm will yield 97% of the dose to 100% of the IPL volume.

Another planning study [9] concludes that even a hypofractionated SIB is feasible but ‘requires small margins needing intrafraction motion tracking’. This is in agreement with our simulation indicating that a safety margin of only around 2 mm might be a good compromise in terms of dose delivery to the IPL and toxicity. From other work [19, 20], we agree that online tracking and correction is the preferred approach to counter intrafraction motion.

However, there was considerable variability between patients. On the one hand, in a patient with little motility, an average displacement of the prostate of only 0.5 mm meant that 2 mm margins would have covered the complete lesion volume with at least 98% of the dose. On the other hand, in a patient with especially high motility, an average displacement of the prostate of 2.5 mm meant that 2 mm margins would have ensured only 72% of the total dose to 100% of the lesion volume. Here even a margin of 5 mm would ensure coverage with 90% of the prescribed dose to 69% of the volume of a lesion of radius 7 mm.

There are a couple of limitations to our study. Foremost, our simulation takes the recorded trajectories as ground truth and does not consider noise on top of them. Realistic margins will take all sources of error into account. The simplest way to do so (albeit not technically correct) would be to treat the intra-fraction motion as one of several sources of variance and simply add those up. The caveat lies in the fact that while other sources of error have constant variance, the variance of intra-fraction increases linearly in time [18, 19]. This leads to non-Gaussian kernels of dose convolution [21]. This also means that standard optimal margin recipes for inter-fractional motion, in so far as they assume a Gaussian distribution of displacements [22], are not perfectly valid for intra-fraction motion.

Also, our simulation does not take any interference between the motion of the apparatus (e.g. collimator leaves) and the motion of the prostate into account. The simulation simply assumes a constant and perfectly spherical dose distribution. We also did not consider any effects of target rotation or deformation. The IPL is assumed to be a constant and perfectly spherical volume. In comparison to more realistic 4D dose reconstruction techniques [14, 23] our approach is a simplification dictated by available data, i.e. tracking of the prostate center only.

Furthermore, an interesting question which is out of the scope of this simulation is the question how much dose a neighboring organ at risk receives due to intra-fraction motion. On the one hand, one could use our model for a simple calculation by looking at two spheres at a certain initial distance. However, a sphere is already an idealization of an IPL and is even less so a suitable proxy for the shape of organs at risk such as e.g. the rectum.

## Conclusion

While sometimes the prostate strayed far from its initial position, on average over all fractions and patients, the dose coverage would have been acceptable even for small target volumes such as IPLs of radius 3 to 7 mm and narrow fields. Moderate safety margins of 2 mm could have ensured a delivery of 90% or more of the SIB dose to the IPL in the average patient. In this case, SIB volume would have been considerably larger than IPL volume, but still considerably smaller than the overall PTV of the prostate. However, there is considerable variability between patients, with some extreme cases requiring larger margins still for a satisfactory dose coverage.

## Additional file

Additional file 1: **Table S5.** DVH, volume in % of IPL volume as a function of dose in % of nominal SIB dose. (DOCX 26 kb)

## Abbreviations

4DUS: 4D ultrasound; ultrasound imaging resolving both volume (3D) and time (1D)
DVH: Dose volume histogram
IPL: Intraprostatic lesion
SIB: Simultaneous integrated boost

## References

[1] Singh AK, Guion P, Sears-Crouse N, Ullman K, Smith S, Albert PS (2007). Simultaneous integrated boost of biopsy proven, MRI defined dominant intra-prostatic lesions to 95 gray with IMRT: early results of a phase I NCI study. Radiat Oncol.

[2] Rischke HC, Nestle U, Fechter T, Doll C, Volegova-Neher N, Henne K (2013). 3 tesla multiparametric MRI for GTV-definition of dominant Intraprostatic lesions in patients with prostate Cancer--an interobserver variability study. Radiat Oncol.

[3] von Eyben FE, Kiljunen T, Kangasmaki A, Kairemo K, von Eyben R, Joensuu T (2016). Radiotherapy boost for the dominant Intraprostatic Cancer lesion-a systematic review and meta-analysis. Clin Genitourin Cancer.

[4] Zamboglou C, Klein CM, Thomann B, Fassbender TF, Rischke HC, Kirste S (2018). The dose distribution in dominant intraprostatic tumour lesions defined by multiparametric MRI and PSMA PET/CT correlates with the outcome in patients treated with primary radiation therapy for prostate cancer. Radiat Oncol.

[5] Pinkawa M, Piroth MD, Holy R, Klotz J, Djukic V, Corral NE (2012). Dose-escalation using intensity-modulated radiotherapy for prostate cancer - evaluation of quality of life with and without (18) F-choline PET-CT detected simultaneous integrated boost. Radiat Oncol.

[6] Sundahl N, De Meerleer G, Villeirs G, Ost P, De Neve W, Lumen N (2016). Combining high dose external beam radiotherapy with a simultaneous integrated boost to the dominant intraprostatic lesion: analysis of genito-urinary and rectal toxicity. Radiother Oncol.

[7] Monninkhof EM, van Loon JWL, van Vulpen M, Kerkmeijer LGW, Pos FJ, Haustermans K (2018). Standard whole prostate gland radiotherapy with and without lesion boost in prostate cancer: toxicity in the FLAME randomized controlled trial. Radiother Oncol.

[8] Timon G, Ciardo D, Bazani A, Marvaso G, Riva G, Volpe S (2018). Short-term high precision radiotherapy for early prostate cancer with concomitant boost to the dominant lesion: ad interim analysis and preliminary results of phase II trial AIRC-IG-13218. Br J Radiol.

[9] Tree A, Jones C, Sohaib A, Khoo V, van As N (2013). Prostate stereotactic body radiotherapy with simultaneous integrated boost: which is the best planning method?. Radiat Oncol.

[10] Abdellatif A, Craig J, Jensen M, Mulligan M, Mosalaei H, Bauman G (2012). Experimental assessments of intrafractional prostate motion on sequential and simultaneous boost to a dominant intraprostatic lesion. Med Phys.

[11] Sihono DSK, Ehmann M, Heitmann S, von Swietochowski S, Grimm M, Boda-Heggemann J, Lohr F, Wenz F, Wertz H (2018). Determination of Intrafraction prostate motion during external beam radiation therapy with a Transperineal 4-dimensional ultrasound real-time tracking system. Int J Radiat Oncol Biol Phys.

[12] Keall PJ, Aun Ng J, O'Brien R, Colvill E, Huang CY, Rugaard Poulsen P, Fledelius W, Juneja P, Simpson E, Bell L, Alfieri F, Eade T, Kneebone A, Booth JT (2015). The first clinical treatment with kilovoltage intrafraction monitoring (KIM): a real-time image guidance method. Med Phys.

[13] Rosario T, van der Weide L, Admiraal M, Piet M, Slotman B, Cuijpers J (2018). Toward planning target volume margin reduction for the prostate using intrafraction motion correction with online kV imaging and automatic detection of implanted gold seeds. Pract Radiat Oncol.

[14] Colvill E, Booth JT, O'Brien RT, Eade TN, Kneebone AB, Poulsen PR, Keall PJ (2015). Multileaf collimator tracking improves dose delivery for prostate Cancer radiation therapy: results of the first clinical trial. Int J Radiat Oncol Biol Phys.

[15] Murtaza G, Toftegaard J, Khan EU, Poulsen PR (2017). Volumetric modulated arc therapy with dynamic collimator rotation for improved multileaf collimator tracking of the prostate. Radiother Oncol.

[16] Fast MF, O'Shea TP, Nill S, Oelfke U, Harris EJ (2016). First evaluation of the feasibility of MLC tracking using ultrasound motion estimation. Med Phys.

[17] Ipsen S, Bruder R, O’Brien R, Keall PJ, Schweikard A, Poulsen PR (2016). Online 4D ultrasound guidance for real-time motion compensation by MLC tracking. Med Phys.

[18] Ballhausen H, Li M, Ganswindt U, Belka C (2018). Shorter treatment times reduce the impact of intra-fractional motion : a real-time 4DUS study comparing VMAT vs. step-and-shoot IMRT for prostate cancer. Strahlenther Onkol.

[19] Ballhausen H, Li M, Hegemann NS, Ganswindt U, Belka C (2015). Intra-fraction motion of the prostate is a random walk. Phys Med Biol.

[20] Ballhausen H, Ganswindt U, Belka C, Li M (2016). Intra-fraction motion of the prostate is not increased by patient couch shifts. Radiat Oncol.

[21] Ballhausen H, Reiner M, Kantz S, Belka C, Söhn M (2013). The random walk model of intrafraction movement. Phys Med Biol.

[22] van Herk M, Remeijer P, Lebesque JV (2002). Inclusion of geometric uncertainties in treatment plan evaluation. Int J Rad Onc Biol Phys.

[23] Poulsen PR, Schmidt ML, Keall P, Worm ES, Fledelius W, Hoffmann L (2012). A method of dose reconstruction for moving targets compatible with dynamic treatments. Med Phys.

