# Supplementary material for: Dosimetric impact of intrafraction motion on boosts on intraprostatic lesions: a simulation based on actual motion data from real time ultrasound tracking
Source: Radiat Oncol. 2019 May 16;14:81. doi: 10.1186/s13014-019-1285-1 (PMC6524311; doi:10.1186/s13014-019-1285-1)
Supplement: Supplementary file 1 — Table S5. DVH, volume in % of IPL volume as a function of dose in % of nominal SIB dose. (DOCX 26 kb) [file 13014_2019_1285_MOESM1_ESM.docx]

**Table S5 – DVH, volume in % of IPL volume as a function of dose in % of nominal SIB dose**

| Safety margin | none |  |  | 2 mm |  |  | 5 mm |  |  |
| --- | --- | --- | --- | --- | --- | --- | --- | --- | --- |
| IPL radius | 3 mm | 5 mm | 7 mm | 3 mm | 5 mm | 7 mm | 3 mm | 5mm | 7mm |
|  |  |  |  |  |  |  |  |  |  |
| 30% of dose | 100% | 100% | 100% | 100% | 100% | 100% | 100% | 100% | 100% |
| 35% of dose | 99% | 100% | 100% | 100% | 100% | 100% | 100% | 100% | 100% |
| 40% of dose | 97% | 100% | 100% | 100% | 100% | 100% | 100% | 100% | 100% |
| 45% of dose | 94% | 98% | 99% | 100% | 100% | 100% | 100% | 100% | 100% |
| 50% of dose | 91% | 96% | 98% | 100% | 100% | 100% | 100% | 100% | 100% |
| 55% of dose | 86% | 94% | 96% | 100% | 100% | 100% | 100% | 100% | 100% |
| 60% of dose | 80% | 91% | 94% | 100% | 100% | 100% | 100% | 100% | 100% |
| 65% of dose | 73% | 87% | 91% | 100% | 100% | 100% | 100% | 100% | 100% |
| 70% of dose | 65% | 82% | 88% | 100% | 100% | 100% | 100% | 100% | 100% |
| 75% of dose | 54% | 76% | 84% | 100% | 100% | 100% | 100% | 100% | 100% |
| 80% of dose | 39% | 68% | 78% | 100% | 100% | 100% | 100% | 100% | 100% |
| 85% of dose | 21% | 55% | 70% | 100% | 100% | 100% | 100% | 100% | 100% |
| 90% of dose | 1% | 37% | 56% | 100% | 100% | 100% | 100% | 100% | 100% |
| 95% of dose | 0% | 9% | 31% | 42% | 83% | 90% | 100% | 100% | 100% |
| 99% of dose | 0% | 0% | 0% | 0% | 0% | 12% | 7% | 58% | 74% |

Percentage of the IPL volume that is receives a given percentage of the SIB nominal dose. Unweighted statistics of 39,295 recorded positions during 720 fractions in 28 patients.
